# IL-18: The Forgotten Cytokine in Dengue Immunopathogenesis

**DOI:** 10.1155/2021/8214656

**Published:** 2021-11-19

**Authors:** Josephine Diony Nanda, Tzong-Shiann Ho, Rahmat Dani Satria, Ming-Kai Jhan, Yung-Ting Wang, Chiou-Feng Lin

**Affiliations:** ^1^International Ph.D. Program in Cell Therapy and Regenerative Medicine, College of Medicine, Taipei Medical University, Taipei 110, Taiwan; ^2^Department of Pediatrics, National Cheng Kung University Hospital, College of Medicine, National Cheng Kung University, Tainan 704, Taiwan; ^3^Department of Pediatrics, Tainan Hospital, Ministry of Health and Welfare, Tainan 700, Taiwan; ^4^International Ph.D. Program in Medicine, College of Medicine, Taipei Medical University, Taipei 110, Taiwan; ^5^Department of Clinical Pathology and Laboratory Medicine, Faculty of Medicine, Public Health and Nursing, Universitas Gadjah Mada, Yogyakarta 55281, Indonesia; ^6^Clinical Laboratory Installation, Dr. Sardjito Central General Hospital, Yogyakarta 55281, Indonesia; ^7^Department of Microbiology and Immunology, School of Medicine, College of Medicine, Taipei Medical University, Taipei 110, Taiwan; ^8^Graduate Institute of Medical Sciences, College of Medicine, Taipei Medical University, Taipei 110, Taiwan

## Abstract

Dengue fever is an infection by the dengue virus (DENV) transmitted by vector mosquitoes. It causes many infections in tropical and subtropical countries every year, thus posing a severe disease threat. Cytokine storms, one condition where many proinflammatory cytokines are mass-produced, might lead to cellular dysfunction in tissue/organ failures and often facilitate severe dengue disease in patients. Interleukin- (IL-) 18, similar to IL-1*β*, is a proinflammatory cytokine produced during inflammation following inflammasome activation. Inflammatory stimuli, including microbial infections, damage signals, and cytokines, all induce the production of IL-18. High serum IL-18 is remarkably correlated with severely ill dengue patients; however, its possible roles have been less explored. Based on the clinical and basic findings, this review discusses the potential immunopathogenic role of IL-18 when it participates in DENV infection and dengue disease progression based on existing findings and related past studies.

## 1. Introduction

Dengue disease is a primary *Flaviviridae* infection worldwide caused by the dengue virus (DENV) [1, 2]. DENV comprises four different serotypes (DENV1 to 4), with a wide range of genotypes and variants [3]. This myriad of DENV serotypes and variants are hypothesized to mediate its survival, together with increasing infectivity [4]. DENV infects humans as the primary host, transmitted via mosquitos mainly in tropical and subtropical areas [5]. Yearly, DENV is predicted to infect 100–400 million people worldwide [1]. Even though in 2021, the DENV infection incidence and mortality rate are reduced compared to 2020, the infection is still spread in many areas, increasing the health burden in this COVID-19 pandemic era [6]. Symptoms of the dengue diseases are widely varied. It could be shown as mild flu-like symptoms, mild dengue fever (MDF), to severe symptoms, the severe dengue diseases (SDDs,) in those who are infected. In MDF, the common symptom found is fever accompanied by one of the following: nausea, vomiting, rash, aches, and pains. Dengue hemorrhagic fever (DHF) and dengue shock syndrome (DSS) are two types of severe dengue. In addition, multiple organ dysfunction and central nervous system (CNS) impairment are also involved in SDDs. Although rare, severe dengue can result in a variety of consequences, including excessive bleeding, organ damage, plasma leakage, and even death [1, 7–9].

## 2. Dengue Pathogenesis

Virus factors and host response majorly influence dengue severity. The variance in dengue serotype provides them numerous possibilities in causing severe DENV infection. As one of the oldest strains known, DENV-2 is more prevalent in causing severe dengue (DHF/DSS) and epidemics than other serotypes [4, 10, 11]. However, in several areas, DENV serotypes inducing severe infection started to shift to DENV-1, as reported in Singapore [12] and Indonesia [13]. Regarding the different subtypes of DENV, the American subtype is less likely to cause DHF/DSS than the Asian subtype. It might be facilitated by the higher replicability of the Asian subtype in the *Aedes aegypti* mosquitoes, enhancing their transmission [14, 15]. The DENV genetic variance also influences the intensity of the infection. For example, the difference in E-390 amino acid affects DENV virulence and survival, as it determines the virus's ability to infect and replicate in monocyte-derived macrophages [16]. The sequence of the 3′ untranslated region (UTR) also influences DENV virulence [17]. Other reports demonstrated that higher monocyte infectivity is associated with its ability to generate severe infections together with higher transmission [18].

More personalized factors influencing severe DENV infection, the host factors, are commonly found in secondary heterologous DENV infection, which causes antibody-dependent enhancement (ADE). This event is related to the inability of the previous dengue antibodies to neutralize the recent heterologous DENV infection, allowing easy access of the virus to infect the Fc-presenting cells. This will result in increased viral replication and severe infection [19–21]. Such cases can be observed in Peru, where homologous virus and American DENV-2 virus were neutralized far more efficiently by sera with DENV-1 antibody than Asian DENV-2 viruses [22]. Another situation found in Havana shows that the infection sequence also influences severity. In DENV-1 followed by DENV-3 (DENV-1/DENV-3), infection was linked to severe disease, but DENV-2/DENV-3 was linked to mild/asymptomatic infections. Interestingly, secondary infection also has higher genetic variability compared to the primary one. In DENV-1/DENV-3 secondary infection, changes in premembrane (PrM) and envelope (E) structural proteins might represent the DENV evolution to more potent strain overtimes [23]. This might explain the point regarding the infection incidence in serotype switch dengue epidemics [24].

## 3. Cytokine Response in DENV Infection

Cytokine storm, also called cytokine release syndrome (CRS), is an umbrella term describing several severe symptoms caused by systemic inflammatory syndromes encompassing an increase in blood cytokine levels and hyperactivation of immune cells. This condition may be caused by various pathogens, cancer, autoimmunity, and treatments that activate false alarms, leading to the hyperactivating immune system. Various factors affected cytokine storm incidences, such as genetics (improper inflammasome activation), an inappropriate or inadequate immune response involving activation of effector cells, an overwhelming viral burden, uncontrolled infection that causes prolonged immune stimulation, and the inability to resolve the immune response and revert to homeostasis. Negative feedback mechanisms that are supposed to prevent hyperinflammation and the overproduction of inflammatory cytokines and soluble mediators fail in each of these situations, leading to multiorgan damage [25]. Even though only a few reports regarding cytokine storms in flavivirus infections have been published, some infections, such as DENV [26], Zika virus (ZV) [27], West Nile virus (WNV) [28], Yellow fever virus (YFV) [29], and Japanese encephalitis virus (JEV) [30], are capable of causing cytokine disturbances that lead to poor patient outcomes.

In DENV infection, cytokine storms have been proposed to correlate with inflammasome activation [26, 31, 32]. DENV infected cells *in vivo* and *in vitro* are reported to have higher NLRP3-inflammasome activation via nonstructural (NS)2A and NS2B proteins induction [33], especially in mouse bone marrow-derived macrophages (BMDMs), endothelial cells, keratinocytes, platelets, dendritic cells (DCs), human peripheral blood mononuclear cells (PBMCs), and monocyte-differentiated macrophages (THP-1) [32]. Inflammasome activation also can be induced from reactive oxygen species (ROS) levels through extracellular signal-regulated protein kinases 1 and 2 (ERK1/2) and mitogen-activated protein kinases (MAPK) which found accumulated in DENV infected DCs. The intracellular ROS build-up has proven essential to influence the innate immune response in DENV clearance and promote mitochondrial apoptosis in infected DCs. Further, inflammasome activation will activate caspase and initiate pyroptosis, a lytic programmed cell death, in the cells. Simultaneously, caspase activation also cleavage the pro-IL-1*β* and pro-IL-18 to their active form, causing the inflammatory cascade to be activated and promoting further advances in dengue pathogenesis [34, 35].

A previous study reported an increase in cytokines, such as tumor necrosis factor- (TNF-) *α*, monocyte chemoattractant protein- (MCP-) 1 (CCL-2), regulated upon activation, normal T cell expressed and presumably secreted (RANTES) (CCL-5), interferon- (IFN-) *γ*, IFN-*γ*-induced protein- (IP-) 10 (CXCL-10), IL-4, IL-6, IL-8 (CXCL-8), IL-10, and granulocyte/macrophage colony-stimulating factor (GM-CSF) (CSF-2), in severe DENV infection [36–38]. Secondary infections mainly cause a cytokine storm in dengue due to the ADE effect, which results in overactivation of the immune system and excessive production of proinflammatory cytokines. However, this spurt of proinflammatory cytokines is not accompanied by proper degranulation functions, leading to ineffective eradication of infected cells [39]. Other evidence from the severe case febrile phase of dengue patients presented a decline in total CD4^+^ T, T helper (Th) 1, and Th17 cells in contrast to the convalescent phase [40], demonstrating why some patients move to recovery after the critical phase and others developed dysregulated cytokine production that led to fatal DENV infection followed by CRS progression.

## 4. The Biological Importance of IL-18

IL-18 is a cytokine previously known as IFN-*γ*-inducing factor (IGIF), firstly discovered in mice with endotoxin shock [41, 42]. Together with IL-1*β* and IL-33, IL-18 is also part of IL-1 family cytokines [43]. IL-18 is produced from immune cells, such as macrophages, Langerhans cells, DCs, and many nonimmune cells, such as osteoblasts, chondrocytes, endothelial cells, keratinocytes, and intestinal epithelial cells (Table 1) [44–51]. IL-18 and IL-1*β* are produced as inactive precursors activated via caspase cleavage, generally in an inflammasome-regulated manner, in the cytoplasm before being released into the bloodstream [52]. This activated form of IL-18 enhances adaptive immune activation by inducing IFN-*γ* production by T cells [53], Th1 polarization [54], cytotoxicity of both T cells and natural killer (NK) cells, and maturation of T, NK, and DCs [55, 56]. In addition, free IL-18 can cause innate immune macrophage activation by inducing polarization and inflammatory and cytokine secretion and can even cause macrophage activation syndrome (MAS) [57]. IL-1*β* itself is also known to induce several types of T cells development that take part in some inflammatory conditions and neutrophil recruitment to the infection site [58, 59].

**Figure 1:**
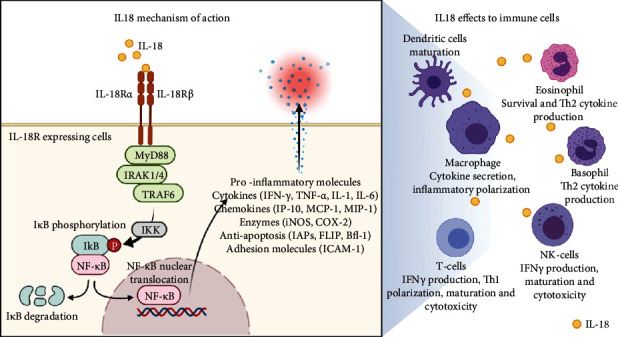
The mechanism of action of IL-18-facilitated inflammatory responses in various immune cells and its possible effects.

IL-18 stimulation is mediated by IL-18 receptors (IL-18R), comprised of the *α* and *β* chains. The binding of IL-18 to IL-18R will relay the signals from myeloid differentiation primary response 88 (MyD88), a primary adapter protein for many TLR and IL-1R family members [60], to IL-1 receptor-associated kinase- (IRAK-) 1/4. Furthermore, IRAK-1/4 catalyzes the ubiquitination of TNF receptor-associated factor- (TRAF-) 6, leading to the activation of I*κ*B kinase (IKK). This kinase will degrade I*κ*B-NF-*κ*B complexes in the cytoplasm, facilitating NF-*κ*B nuclear translocation. This translocation will promote increased expression of various inflammatory cytokines [61], as summarized in Figure 1. Other inflammatory diseases have already proven the mitogen-activated protein kinase (MAPK) pathway involvement by IL-18R receptor activation; however, the role of this mechanism in flavivirus infection is still unknown.

Furthermore, the presence of other cytokines, such as IL-12 or IL-2, enhances the effect of IL-18 in immune cell activation. For example, together with IL-12, IL-18 promotes IFN-*γ* production from Th1 and B cells. Meanwhile, in NK cells, IL-18 alone is enough to cause IFN-*γ* production [53]. However, in an *in vivo* study, IL-12 and IL-18 were essential for maintaining NK cell activity and the Th1 response in bacterial stimulation [62]. In peripheral blood mononuclear cells (PBMCs) treated with IL-18 and IL-2, there was an increase in cytolytic activity, cell proliferation, and IFN-*γ* secretion. The isolated culture of NK cells showed higher proliferation and cytotoxicity activity in the presence of IL-18 and IL-2 compared to T cells [63]. In Th17 cells, IL-18 synergizes with IL-23 and amplifies IL-17 production via T cell receptor (TCR) activation [64]. The exciting part is that IL-18 not only induces Th1 cytokine production but is also capable of activating the humoral immune response via Th2 cytokine production. This phenomenon was first examined in mast cells and basophils cultured with IL-3, a factor required for hematopoietic proliferation and survival, exhibiting high IL-18R*α* expression. Furthermore, stimulation with IL-18 and IL-3 induced massive production of IL-4 and IL-13. However, in the presence of IFN-*γ* and IL-12, the production of IL-4 and IL-13 from mast cells and basophils was highly suppressed [65]. Similar to basophils, treatment of NK and T cells harvested from IFN-*γ* knockout mice with IL-2 and IL-18 showed higher IL-13 mRNA expression than that of cells harvested from wild-type mice [66]. Also, IL-18, via MAPKs, including extracellular signal-regulated kinase (ERK) and p38 MAPK, and NF-*κ*B activation, increases eosinophil survival and the production of IL-6, CXCL8, and CCL2 [67]. More discoveries from Yoshimoto et al. showed that along with IL-4, IL-18 promotes higher IgE production from CD4 T cells, and stimulation of TCR along with IL-18 boosts the differentiation of naïve CD4 T cells to IL-4-producing cells *in vitro* [68]. This complex interplay between cytokines suggests a broad role of IL-18 in determining the host cellular or humoral immune response.

## 5. IL-18 in DENV Infection

The first report about an IL-18 increase in a dengue patient clinical study was published in 2001, where the results from serum examination showed high IL-13 and IL-18 in the severe illness and late dengue disease phase (over 9 days from disease onset) patients [69]. A similar result was obtained from children's cases in Venezuela. It was demonstrated that the IL-18 level was higher in dengue than in control. Moreover, the increase in IL-18 was not associated with NS1 or the infection type (primary or secondary) [70]. Our current report also showed a step ladder increase of IL-18 in severe DENV infection without and with comorbidity (hypertension and or diabetes) to the mild one. The correlation study also found a negative association between platelet and IL-18 level [71]. However, the induction of thrombocytopenia caused by aberrant expression of IL-18 and its possible pathogenic regulation needs further investigation.

The possible mechanism of the IL-18 increase in DENV infection is related to the presence of inflammatory macrophages. This was explained in an *in vitro* study using GM-CSF-induced macrophages (GM-M*ϕ*s). In GM-M*ϕ*s (CD14^+^) primary culture, DENV infection triggers NLRP3 inflammasome activation to cleavage pro-caspase 1 into caspase 1. Further, caspase-1 induces the maturation of pro-IL-1*β* and pro-IL-18, resulting in higher IL-1*β* and IL-18 production from GM-M*ϕ* [72]. The less mature form of macrophage, the monocyte, especially those expressing CD14^+^ or CD16^+^ markers, also secretes IL-18 which causes T-cell activation also IFN-*γ* secretion. However, this IFN-*γ* production is independent of monocyte presence [73].

**Figure 2:**
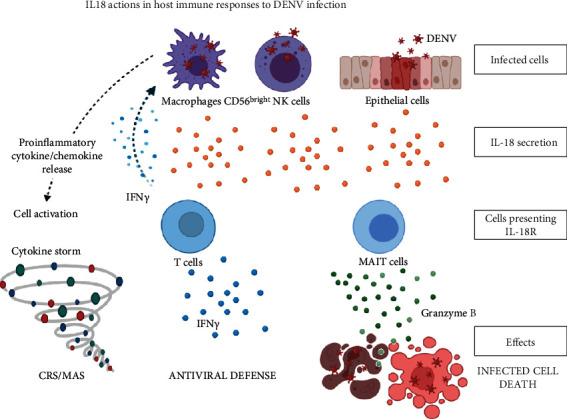
The actions of IL-18 in host immune responses to DENV infection.

IFN-*γ* producing NK cells are also being activated by DENV-induced IL-18 presence. During this event, the less mature NK cells will proliferate and prime to the skin to invade DENV [74]. Apart from those cells, activation of mucosal-associated invariant T (MAIT) cells was also reported following DENV infection. This activation was independent of TCR for cytokine release or Granzyme B upregulation, but it is dependent on IL-18 or in combination with IL-12, IL-15, and/or IFN-*α*/*β*. However, IL-18 levels and MAIT cell activation are linked to infection severity [75]. This peaked increase of IL-18 level might represent the severe patient condition where the inflammation is high. Despite the high level of inflammation, it is not always in line with the ability to eliminate the pathogens, risking it for producing a more severe cytokine response or CRS. In summary, according to the current studies related to DENV-induced IL-18, the possible effects of IL-18 on DENV infection, including cytokine storm, CRS/MAS, antiviral defense, and immune clearance, are summarized in Figure 2.

## 6. Potential Role of IL-18 in Flavivirus Infection and Other Diseases

Although the significance of IL-18 in aiding dengue illness progression is unknown, it has been observed that IL-18 production is changed in metabolic syndromes [76, 77], hypertension [78], diabetic patients [79], cardiovascular disorders [80], atherosclerosis [80, 81], and also several flavivirus infections such as JEV [82], tick-borne encephalitis virus (TBEV) [83], and ZV infection [84, 85]. This increase implies that the presence of IL-18 might play a role as either a protective or pathological cytokine to the host. The CSF of TBEV patients contains several proinflammatory cytokines, including IL-18, and it has a higher concentration of IP-10 (CXCL10), a T cell chemoattractant, than serum [86]. Furthermore, IL-18 is known to induce IFN-*γ* secretion from NK cells, despite suppressing NK cell function in TBEV infection [83]. In ZV infection, an increase in IL-18 levels is also found in pregnant women with fetal development anomalies and infants with CNS deformities [84]. In an *in vivo* model of JEV-infected mice, the expression of IL-1*β* and IL-18 was increased in the brain. When these cytokines are used to treat human microglia (CHME3) and astroglial (SVG) cell lines, increased secretion of proinflammatory cytokines is observed [82]. In contrast, *in vitro* WNV infection modeling does not show any increase in IL-18 production from infected human primary DCs [87] or the transformed human neuroblastoma cell line SK-N-SH [88]. Although there was no increase in IL-18 in response to WNV infection, the NOD-, LRR-, and pyrin domain-containing protein- (NLRP-) 3 inflammasome and IL-1*β* play vital roles in WNT-infected mouse survival. An increased viral load was also found in NLRP3-deficient mice [89]. In Table 2, we summarized IL-18 production in all flavivirus infections and its possible regulation of immune responses. However, most studies are clinical association studies and animal models of infection. No mechanistic investigations have been published.

In the atopic dermatitis mouse model, the knockout of IL-18 reduces skin lesion formation [90]. It means the involvement in Th2-cytokine production and major cytokine plays a role in an allergy reaction. Previously, it has been reported that in DHF, there are shifts of cytokine from Th1 to Th2 type [91], implying its possible role in causing severe dengue progression. Also, IL-18 is one of the cytokines that induce DM patient progression to nephropathy [92]. In chronic obstructive pulmonary disease (COPD) patients, smokers and the end stage of COPD has higher serum level of IL-18 in those who were not smoking and lower stage [93]. Compared to stable, asymptomatic plaques in atherosclerotic patients, unstable plaques had considerably more significant levels of IL-18 mRNA [94]. These two roles of IL-18 in COPD and atherosclerotic patients might indicate the role of IL-18 as a proinflammatory cytokine, worsening the condition of the disease. The IL-18 role in cancer is explained as a dual-edge sword, as its secretion of IFN-*γ* acts as an antitumor mechanism. However, in some cancer polymorphisms, IL-18 correlated with protumoral effects and upregulated VEGF and SD-44 that facilitate metastasis [95]. In triple-negative breast cancer, tumor-derived IL-18 has also been reported to increase PD-1 expression on immunosuppressive NK cells [96], facilitating the immune evasion of the cancer cells. The possible regulation of IL-18 in nonviral human diseases is summarized in Table 3.

## 7. Conclusions

The role of IL-18 in immunomodulating the antiviral response has been studied not only in DENV but also in other diseases. However, in the specific circumstances of high viral burden that cause a lot of infected cell pyroptosis, high levels of IL-18 were secreted, promoting immune overactivation and contributing to the further immunopathogenesis of DENV infection. Together with this understanding, suppressing the activity or production of IL-18 in severely infected patients might prevent the immune overactivation, thus avoiding more severe progression of the disease.

## Figures and Tables

**Table 1:** IL-18 producing cells and the effects after production.

Cells	Study	Origin	Treatment/disease	Types	Potential role	Ref.
Monocytes Macrophages	*Ex vivo*	Mouse liver	Lipopolysaccharide	mRNA	T cell proliferation Spleen cell viability Liver cell injury	[41]
	*Ex vivo*	Human PBMC	Hydroxyapatite	mRNA	Not checked	[51]
Kupffer cells	*Ex vivo*	Mouse liver	—	mRNA	T cell proliferation Spleen cell viability Liver cell injury	[41]
Dendritic cells	*Ex vivo* *In vitro*	Bone marrow LC-like XS 52	GM-CSF and IL-4	RNA protein	Th1 differentiation	[49]
	*Ex vivo*	Human PBMC	IL-4, IL-6, and TNF-*α* Flt3 ligand and GM-CSF	mRNA	Not checked	[50]
Peripheral blood progenitor cells (CD34^+^)	*Ex vivo*	Human PBMC	—	mRNA	Not checked	[50]
Purified blood monocytes (CD14^+^)	*Ex vivo*	Human PBMC	—	mRNA	Not checked	[50]
Lymphoid aggregates and lymphoid follicles	Clinical sample	Intestinal tissue	—	Protein	Cytokine production in T cells	[48]
Osteoblasts	*Ex vivo*	Bone marrow Spleen	—	mRNA	Cell differentiation	[46]
Keratinocyte	Clinical sample	Skin biopsy	—	Protein	Not stated	[47]
Pancreatic *β* cells	Clinical sample	Pancreas	Type 1 diabetes	Protein	Metabolic control	[97]

**Table 2:** IL-18 production in flavivirus infection and its immune responses.

Infection	Source	Host	Level	Immune response	Ref.
DENV	Blood	Human	▲	The increase of IL-18 to detectable levels in the DENV infection febrile phase was significant, which further diminished in the defervescent phase. TNF-*α*, IFN-*γ*, and IL-18 plasma levels also correlated negatively with CD14^high^ CD16^+^ monocytes.	[98]
DENV	Blood	Human	▲	IL-18, TGF-*β*1, and sICAM-1 were increased in severe dengue relative to the mild, accompanied by higher activation makers of T lymphocytes. IL-18 correlated positively with CD8 T cells expressing HLA class-II, CD8 T cells expressing ICAM-1, and plasma ICAM-1.	[99]
DENV	Blood	Macaque	▲	High IFN-*γ*, IL-18, and IL-10 levels together with decreased IL-12 were found in the severe DENV infection of vaccinated macaques. Meanwhile, a slight increase of IL-12 together with IL-18 and no increase of IFN-*γ* and IL-10 were found in the protected macaques.	[100]
DENV	Blood Spleen	Murine	▲	Together with IFN-*γ* and IL-12, IL-18 prevents DENV infection progression to severe and preventable death in the infected mice.	[101]
DENV	Serum Tissue	Murine	—	Together, IL-12 and IL-18 induce IFN-*γ* production and maintain nitric oxide-synthase 2 (NOS2) expression in the spleen, a major regulator in DENV infection control. Diminished IL-12 and IL-18 cause more severe thrombocytopenia and hemoconcentration. Meanwhile, the absence of IL-18 increases the risk of hemoconcentration, liver injury, and a higher viral load leading to higher mortality.	[102]
DENV	Blood	Human	—	The IFN-*γ* response from MAIT cells to DEN and ZV was partially reduced by blocking antibodies against IL-12 and IL-18 and was completely blocked when they were used in combination	[103]
DENV	Blood	Human	▲	DENV infection in the presence of type I IFN and IL-18 increases IFN-*γ* secretion also cytolytic function from primary *γ*ɗ T cells in a TCR-independent manner, but not IL-18R.	[73]
DENV	Blood	Human	▲	DENV infections induce IL-18 and ferritin levels along with the severity, not related to NS1 level and type of infection (primary or secondary).	[70]
DENV	Blood	Human	▲	IL-18 levels were increased early in the febrile phase (days 2-3) of no hyperferritinemia patients; meanwhile, they increased later in the critical phase (days 4-5) in patients with hyperferritinemia compared to other febrile illnesses (OFI).	[104]
DENV	Blood	Human	▲	IL-18 has positive significant association with SGOT and SGPT levels in dengue-infected patients.	[105]
DENV	Cells	Human	▲	Dengue induces inflammasome activation via CLEC5A; Syk-associating receptors in GM-Mɸ cells, not M-Mɸ, further induces IL-1*β* and IL-18 secretion. Dengue-infected GM-Mɸ secretes higher IL-18 compared to M-Mɸ; meanwhile, M-Mɸ secretes higher IL-1*β* to GM-Mɸ.	[72]
DENV	Blood	Human	~	No significant difference in IL-18 gene expression of symptomatic patients to asymptomatic patients.	[106]
DENV	Blood	Human	▲	Positive correlation in serum level of IL-18 and transaminase level.	[107]
DENV	Blood	Human	▲	DENV infection severity (dengue with warning signs and severe dengue) was significantly associated with IL-18 elevation in the febrile and defervescence phase. IL-18 can also be used as predictors for severe DENV infection progression (AUC = 0.768, *P* < 0.0001).	[108]
DENV	Blood	Human	▲	IL-18 promotes less mature NK-cell proliferation and skin-homing in acute DENV infections.	[74]
DENV	Cells	Human	▲	Dengue-infected monocyte cultures showed profound DENV2 replication and higher antiviral cytokine levels (IFN-*α*/*β*, TNF-*α*, IL-12, and IL-18).	[109]
DENV	Cells	Human	▲	DENV infection induces an increase of IFN*α*/*β*, TNF-*α*, IL-12, and IL-18 in monocyte cultures at 24 hour postinfection. Blockade of TIR-domain-containing adapter-inducing interferon-*β* (TRIF), myeloid differentiation primary response (MYD88), or NF-*κ*B suppresses the secretion of these parameters.	[110]
ZV	Blood	Human	▲	Higher IL-18, IL-8, IL-4, IL-22, IL-23, IL-27, MCP-1, TNF-*α*, IP-10, EGF, eotaxin, and FGF-2 in pregnant women correlate with fetal development anomalies. Congenital CNS defect in infants also has higher IL-18 and IP-10 and lower HGF than healthy infants born from ZIKV-infected mothers.	[84]
ZV	Cells	Human	▲	Acute ZIKV infection increases transcripts of IL-1 and IL-18 in monocytes, together with inflammasome involved proteins and caspase 1 and 8 upregulation.	[85]
ZV	Cells	Human	~	Zika infection did not induce pro-IL-1*β*, and pro-IL-18 mRNA increases and was confirmed to have similar IL-1*β* and IL-18 levels in infected astrocytes and mock.	[111]
ZV	Blood	Murine	▲	Zika virus enhanced systematic levels of IFN-*γ* and IL-18 throughout infection.	[112]
ZV	Tissue	Human	▲	Higher expression of inflammasomes, caspase-1, iNOS, arginase-1, IL-33, IL-18, and IL-1*β* in the microcephalic brain compared to the control.	[113]
JEV	Tissue	Murine	▲	JEV infected mice secrete mature Il-18 in a time-dependent manner with a peak level on day 7 postinfection. Replicating JEV induces inflammasome activation and further initiates caspase-1 activation and induces IL-1*β* and IL-18 production.	[114]
JEV	Tissue	Murine	▲	JEV induces upregulation of IL-18 and IL-1*β* in the brain by increased production from microglia and astrocytes. Furthermore, IL-18 and IL-1*β* separately promote cytokine (IL-1*β*, IL-6, IL-8, IL-18, and TNF-*α*) and chemokine (IP-10, MCP-1, MIG, and RANTES) production from microglia and astrocytes. IL-18 or IL-1*β* activated microglia also have higher neurotoxicity in JEV infections.	[82]
WNV	Spleen	Murine	▲	Splenic M*Φ* takes an important role in suppressing WNV infection in Mɸ, monocytes, and splenic CD11c^+^CD11b^−^ DCs by increasing the expression of cytokine (IL-18), complement protein (C1q), the apoptotic cell clearance protein (Mertk), and caspase-12.	[86]
WNV	Cells	Human	~	WNV infection-induced DC secretion of type I interferon (IFN), but no or minimal interleukin (IL) 212, IL-23, IL-18, or IL-10.	[115]
TBEV	Blood	Human	▲	Human TBEV infection induces the increase of NK cell activation together with higher IL-12, IL-15, IL-18, IFN-g, and TNF levels in plasma. Even though in acute infection NK cell function was suppressed, IFN-*γ* producing capacity in IL-12/IL-18 presence was not affected.	[83]
TBEV	CSF Serum	Human	▲	Cerebro-spinal fluid (CSF) of TBE patients had an increase in CXCL10, CXCL11, p40 subunit of IL-12/23, IL-15, and IL-18 levels.	[86]
YFV	Plasma	Human	—	Induction with IL-12 alone, IL-12 and IL-18 or K562 cells in YFV infected NK cells cause more degranulation and IFN-*γ* production.	[116]

▲: increase; ▼: decrease; ~: no changes; —: not explained.

**Table 3:** IL-18 role in other diseases.

Diseases	Effects/condition	Reference
Atopic dermatitis	Induce skin lesion	[90]
Diabetes mellitus	Neuropathy progression	[92]
COPD	Higher in severe	[93]
Atherosclerosis	Higher in severe	[94]
Cancer	Dual role: antitumor, facilitate metastasis and immune evasion	[95, 96]

## Data Availability

The data used to support the findings of this study are available from the corresponding author upon request.
